# Twenty-year changes of penta-chlorodibenzofuran (PeCDF) level and symptoms in Yusho patients, using association analysis

**DOI:** 10.1186/1756-0500-3-129

**Published:** 2010-05-05

**Authors:** Shinya Matsumoto, Yoshiyuki Kanagawa, Soichi Koike, Manabu Akahane, Hiroshi Uchi, Satoko Shibata, Masutaka Furue, Tomoaki Imamura

**Affiliations:** 1 Department of Planning Information and Management, The University of Tokyo Hospital, Tokyo, Japan; 2 Department of Public Health, Health Management and Policy, Nara Medical University School of Medicine, Kashihara, Japan; 3 Department of Dermatology, Graduate School of Medical Sciences, Kyushu University, Fukuoka, Japan

## Abstract

**Background:**

Recently, methods for measurement of dioxins in the blood have improved. Also, techniques for analyzing large quantities of data have been developed, such as data mining. Even in subjects with elusive characteristics, it is becoming possible to find previously unknown characteristics by checking all combinations of symptoms.

**Findings:**

Using association analysis of the data mining technique, we extracted and compared combinations with a strong relationship between recent symptoms (2001-2004) and recent blood PeCDF levels, and between past symptoms (1986-1989) and recent PeCDF levels, in physical, blood, dermatological, dental and ophthalmological examinations.

Patients with a higher PeCDF level were more likely to present with symptoms included in the diagnostic criteria, such as pigmentation. In addition, we obtained evidence that recent PeCDF levels had a stronger relationship with recent than past symptoms.

**Conclusions:**

Recent PeCDF levels should not be compared directly with past symptoms. However, as the excretion rate of PeCDF has been constant, it is probable that PeCDF levels were higher in the past if recent PeCDF levels were also high. The study confirmed a relationship between past PeCDF levels and past clinical symptoms. For symptoms included in the diagnostic criteria, there was a stronger relationship between PeCDF levels and past symptoms than recent symptoms. Alleviation of symptoms in each patient or aging weakened the relationship between PeCDF levels and symptoms.

## Background

Kanemi Yusho is a form of food poisoning that was caused by rice bran oil in 1968 in Western Japan, especially in Northern Kyushu [1]. Initially, polychlorinated biphenyls (PCBs), which are used as a heat medium in the process of rice bran oil manufacture, were considered as the causative agents. However, subsequent research threw suspicion on 2,3,4,7,8-penta-chlorodibenzofurans (PeCDFs), which are dioxins that result from heat-denatured PCB, as causative agents. Today, PCBs and their derivatives are considered to be the main causative agents of Kanemi Yusho [1-5].

With recent advances in measurement techniques for dioxins such as PeCDF, measurement of blood PeCDF levels has become possible using the same amount of blood as that used in the annual examinations. Since the Yusho examinations in 2001, PeCDF measurement has started for patients who wish such an examination [6,7].

A few decades have passed since the occurrence of Kanemi Yusho, and the symptoms at the initial phase have already disappeared. We have reported previously the relationship between clinical symptoms of Yusho patients and dioxins, one of the causative agents of Yusho [8,9]. Until now, collective results that show the relationship between Yusho and its symptoms have been reported [10]. In addition, several reports have compared these results [2]. Direct comparison of the latest and previous reports is difficult because study patients may have differed between the studies. In addition, it is difficult to differentiate between patients' low exposure to PeCDF, when the incident occurred, and amelioration of symptoms over time.

Recently, methods for mass data analysis, e.g. data mining, have improved markedly. In particular, it has become possible to scrutinize and evaluate many combinations of symptoms which had been considered too large to handle previously [11]. This study used data mining to investigate the relationship between recent/past symptoms and PeCDF levels in the same patients, and the relationship between PeCDF levels and symptoms at a time close to the occurrence of Kanemi Yusho. It is currently difficult to distinguish the characteristic Yusho symptoms from aging symptoms. As a result of the long half-life of PeCDF, patients who ingested a large amount of PeCDF at the time of the accident still have high levels of PeCDF, albeit with a gradual reduction over time.

We think that the association between the symptoms and PeCDF level can be examined more properly by using data from the period when the characteristic Yusho symptoms were still evident. This study aimed to confirm the association between PeCDF level and Yusho symptoms, which recently seems to have become obscured, by using historical data.

## Methods

### Subjects and examination items

After the occurrence of Kanemi Yusho, health examinations for designated Yusho patients (hereinafter referred to as Yusho examinations) have been conducted since 1986. We used data on the presence or absence of symptoms in physical, blood, dermatological, dental and ophthalmological examinations in Yusho patients who had undergone annual examinations in 1986-1989 and 2001-2004, at least once in each period, and whose PeCDF levels were measured. Subjects included both designated and undesignated patients. The number of subjects who underwent examinations in both periods was 302. To analyze the relationship with recent PeCDF levels, representative values were obtained for each item in the physical, blood, dermatological, dental and ophthalmological examinations for each year in 1986-1989 and 2001-2004, for each patient. We defined abnormality as one or more abnormal values in 4 years. Yusho examinations consisted of a total of 241 items, including 52 items in a questionnaire, 55 in physical and laboratory examinations, 21 in dermatological examinations, 108 in dental examinations, and five in ophthalmological examinations [1,7].

### Methods for data analysis

We found laboratory parameters that were related strongly to recent PeCDF level among the past and recent data. We used association analysis [12] to identify symptoms that were strongly related to PeCDF level. Association analysis was applied in medical domain [8,13]. The numerical value that indicated the level of association with PeCDF level was compared between the strongly associated past and recent symptoms. The proportion of patients with high PeCDF levels and those with each symptom were calculated. If a high level of PeCDF and the presence of each symptom were not related, the rate of patients with a certain symptom among those with high PeCDF levels was calculated as the product of both proportions, and is binomially distributed. If the sample size was adequate, the binomial distribution could be considered as a normal distribution. The relation of symptom to the PeCDF level was judged based on how the symptom was distributed in the probability distribution. Z-score was used to show the difference from the mean in the normal distribution [8]. Association analysis revealed highly-related combinations of symptoms and PeCDF levels, which were defined as a z score ≥ 1.645 (one-tailed significance level, 5%).

### Classification of examination results

Designation of Yusho was based upon the diagnostic criteria in Additional File 1[1]. The diagnostic criteria largely consist of "onset conditions," "important findings" and "reference symptoms and findings." Among these, severity of "acneform eruptions" and "pigmentation" ("important findings") was determined differently by the doctors who conducted the examinations, whereas measurement of "abnormalities of blood PCB properties and levels," "abnormalities of blood polychlorinated quarterphenyl (PCQ) properties and levels" and "abnormalities of blood PeCDF levels" can be expressed numerically, thereby playing an important role in determining designation.

To conduct association analysis, the numerical data were classified into categorical data such as "within the normal range" and "abnormal values". For general blood examination, standard values that the study group used in examinations were used to define "normal range". Findings from the physical, dermatological, dental and ophthalmological examinations were each classified by the presence or absence of abnormalities, and their strength.

With regard to PCBs, it is known that chromatographic analysis patterns in Yusho patients differ from those in the general population. Patterns are classified into four types: Type A, which is specific to Yusho patients; Type C, which is specific to the general population; Type B, which is intermediate between Types A and C; and Type BC, which is intermediate between Types B and C.

For PCQ levels, it was considered that ≥ 0.1 ppb was abnormally high; ≤ 0.02 ppb (detection limit) was normal; and 0.03-0.09 ppb was borderline. As for PeCDF level, "50 pg/g lipid or above" and < 50 pg/g lipid were classified as high and normal level, respectively.

## Results

Table 1 indicates the recent symptoms that were independently highly related to recent high PeCDF level (≥ 50 pg/g lipid): Type A PCB pattern, past pigmentation, and high uric acid level.

**Table 1:** Recent symptoms with independently high relationship with high PeCDF level (≥ 50 pg/g lipid)

*Symptoms*	*Rate of patients with symptom*	*Rate of patients with high PeCDF*	*Z score*
PCB pattern (A)	0.411	0.774	3.513
Past pigmentation (+)	0.487	0.667	1.901
Uric acid (high)	0.301	0.692	1.755

Table 2 indicates the past symptoms that were highly related to recent high PeCDF level (≥ 50 pg/g lipid): Type A and B PCB pattern, and high uric acid level.

**Table 2:** Past symptoms with independently high relationship with high PeCDF level (≥ 50 pg/g lipid)

*Symptoms*	*Rate of patients with symptom*	*Rate of patients with high PeCDF*	*Z score*
PCB pattern (A)	0.427	0.791	3.891
Uric acid (high)	0.205	0.758	2.135
PCB pattern (B)	0.272	0.707	1.846

Table 3 compares the z score between recent and past symptoms that were highly related to recent high PeCDF level (≥ 50 pg/g lipid). Recent symptoms had a lower z score than past symptoms for Type A PCB pattern and high uric acid level. Type B PCB pattern was more strongly related to past than recent symptoms.

**Table 3:** Comparison of z score of symptoms with high relationship with PeCDF level (≥ 50 pg/g lipid)

	*Past *	*Recent*
PCB pattern (A)	3.891	3.513
Uric acid (high)	2.135	1.755
PCB pattern (B)	1.846	≤ 1.645
Past pigmentation (+)	≤ 1.645	1.901

≤ 1.645 indicates lack of significant association.

Table 4 indicates the recent symptoms that were independently highly related to recent low PeCDF level (< 50 pg/g lipid). Type C PCB pattern showed the strongest relationship, and there was also a strong relationship with odontogenesis imperfecta.

**Table 4:** Recent symptoms with independently low relationship with low PeCDF level (< 50 pg/g lipid)

*Symptoms*	*Rate of patients with symptom*	*Rate of patients with high PeCDF*	*Z score*
PCB pattern (C)	0.185	0.893	5.439
Odontogenesis imperfecta (+)	0.017	1.000	1.930
Hepatomegaly (+)	0.073	0.682	1.795
Urinary protein (abnormal)	0.142	0.605	1.756
Nutrition (thin)	0.023	0.857	1.710
Inorganic phosphorus (low)	0.040	0.750	1.677
Palpebral conjunctiva pigmentation (+ or above)	0.070	0.667	1.646

Table 5 indicates past symptoms that were independently highly related to recent low PeCDF level (< 50 pg/g lipid). Type C PCB pattern showed the strongest relationship, and high albumin level also appeared as a symptom.

**Table 5:** Past symptoms with independently low relationship with low PeCDF level (< 50 pg/g lipid)

*Symptoms*	*Rate of patients with symptom*	*Rate of patients with high PeCDF*	*Z score*
PCB pattern (C)	0.182	0.855	4.937
Smoking state (+)	0.169	0.667	2.623
Albumin (high)	0.046	0.786	2.019
Inferior gingival pigmentation (brown)	0.162	0.612	1.968
Blood glucose (low)	0.033	0.800	1.771
Total bilirubin (high)	0.119	0.611	1.659

Table 6 compares the z score between recent and past symptoms that were highly related to recent low PeCDF level (< 50 pg/g lipid). Recent symptoms had a lower z score than past symptoms for Type C PCB pattern, therefore, it was more strongly related to past than recent symptoms. In addition, odontogenesis imperfecta and hepatomegaly appeared only as past symptoms.

**Table 6:** Comparison of z score of symptoms with low relationship with low PeCDF level (< 50 pg/g lipid)

*Symptoms*	*Past*	*Recent*
PCB pattern (C)	5.439	4.937
Odontogenesis imperfecta (+)	1.930	≤ 1.645
Hepatomegaly (+)	1.795	≤ 1.645
Urinary protein (abnormal)	1.756	≤ 1.645
Nutrition (thin)	1.710	≤ 1.645
Inorganic phosphorus (low)	1.677	≤ 1.645
Palpebral conjunctiva pigmentation (+ or above)	1.646	≤ 1.645
Smoking state (+)	≤ 1.645	2.623
Albumin (high)	≤ 1.645	2.019
Inferior gingival pigmentation (brown)	≤ 1.645	1.968
Blood glucose (low)	≤ 1.645	1.771
Total bilirubin (high)	≤ 1.645	1.659

≤ 1.645 indicates lack of significant association.

**Figure 1:**
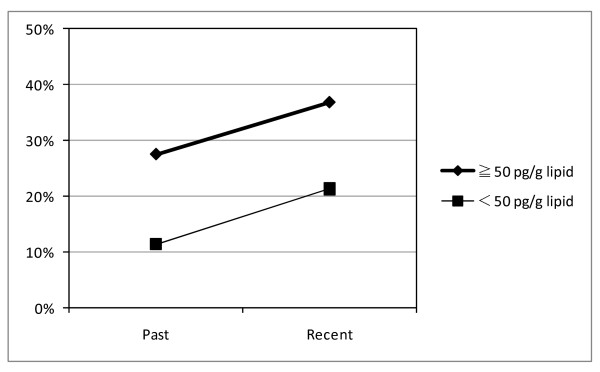
**Prevalence of high level of uric acid**.

Figure 1 shows the prevalence of a high level of uric acid. This was more strongly associated with PeCDF ≥ 50 pg/g lipid than < 50 pg/g lipid, both as a past and recent symptom. The prevalence was increased in patients with PeCDF ≥ 50 pg/g lipid and < 50 pg/g lipid. The odds ratio of prevalence between the PeCDF ≥ 50 pg/g lipid and < 50 pg/g lipid groups was 2.93 in the past. The odds ratio was 2.15 in the recent period.

## Discussion

Type A PCB pattern was strongly related to high PeCDF level (≥ 50 pg/g lipid), and Type C pattern was strongly related to low PeCDF level (< 50 pg/g lipid). In addition, the results showed that Type B PCB pattern was related to high PeCDF level in the past, but there was no such tendency more recently. Specifically, the results suggested a stronger relationship with the past than with the recent PCB pattern. In the past, measuring trace dioxins was difficult. Gas chromatography was used to measure mass per molecular weight as a relative amount. PCB patterns were determined based on peaks, i.e., molecular weights at which relative amounts increase. PCB pattern Type A is known to be specific to Yusho, Type C is found in the general population, and Type B is intermediate between these two. These PCB patterns are presumed to change with time, becoming closer to that of the general population.

In addition, there was a relationship between uric acid and past pigmentation, which was one of the diagnostic criteria. This suggests that there was pigmentation in the past, and recent symptoms are expected to change into past symptoms. With regard to uric acid level, the recent odds ratio is smaller than the past odds ratio. This suggests that the difference in PeCDF level was more strongly associated with uric acid level in the past. No relationship has been found for uric acid in previous studies. Here, the patients who presented with at least one abnormality during the study period were considered to have abnormal measurements. Uric acid is a test item that varies greatly among individuals. A stronger correlation with PeCDF values is found in male than in female subjects. In female subjects, uric acid is more likely to be judged as abnormal because of its small normal range, which may lead to the contingent results. In the present study, to find combinations of symptoms sensitively while symptoms were disappearing, patients were designated as abnormal when they presented with at least one abnormality. The results should be considered in view of the contingency of this report.

In patients with low PeCDF (< 50 pg/g lipid), inferior gingival pigmentation (brown) appeared as a recent but not a past symptom. Development of marked symptoms in patients with lower recent PeCDF levels is unlikely from a medical standpoint, therefore, some other causes are assumed (e.g., pigmentation was determined differently by the doctors who conducted the examinations).

Measurement of PeCDF level has only commenced recently. In the present study, recent PeCDF levels and past symptoms were compared. Fundamentally, there is a problem with direct comparison between recent PeCDF levels and past symptoms, because they are from different times. However, we consider that comparison is valid because the excretion rate of PeCDF has been constant over time [1], and this was a cohort study that allowed identification of each patient in the annual examinations.

## Limitations

There was no direct relationship between past symptoms and recent measurements because of problems with the excretion rate of PeCDF, therefore, the levels should be considered as reference values. In addition, patients might have been exposed to organochlorine compounds between the periods compared: i.e. 1986-1989 and 2001-2004. However, it can be assumed that the level of such exposure would have been far lower than the level that was already present in Yusho patients. Furthermore, there might be other diseases that can develop in association with aging and other factors. It is unlikely that another disease was involved, but we will be able to clarify this by further investigating the symptoms observed in this study.

## Conclusion

In this analysis, we studied the relationship between recent PeCDF levels and recent/past symptoms. The past symptoms in patients with high PeCDF levels were similar to the symptoms described in the diagnostic criteria of Yusho. Patients with recent high PeCDF levels also had past high PeCDF, and a relationship with past symptoms is therefore assumed. Past symptoms were demonstrated to have a stronger relationship with PeCDF level than were recent symptoms. Alleviation of symptoms in each patient, or an increase in symptoms because of aging, was shown to decrease the clarity of symptoms.

## Competing interests

The authors declare that they have no competing interests.

## Authors' contributions

SM designed the study, drafted the manuscript, designed the data analysis, and analyzed the data. YK, SK and MA assisted with drafting the manuscript. HU, SS and MF collected the data. TI designed the whole study and assisted with manuscript drafting. All the authors reviewed the final manuscript and gave their approval.

## Supplementary Material

Additional file 1 **Appendix 1**. Diagnostic criteria for Yusho (as presently supplemented). Click here for file
