# Supplementary material for: Twenty-year changes of penta-chlorodibenzofuran (PeCDF) level and symptoms in Yusho patients, using association analysis
Source: BMC Res Notes. 2010 May 5;3:129. doi: 10.1186/1756-0500-3-129 (PMC2880124; doi:10.1186/1756-0500-3-129)
Supplement: Additional file 1 — Appendix 1. Diagnostic criteria for Yusho (as presently supplemented). [file 1756-0500-3-129-S1.DOC]

**Appendix 1** Diagnostic criteria for Yusho (as presently supplemented)

| **The diagnostic criteria for Yusho were revised on October 26, 1972; supplemented on June 14, 1976; and an item related to blood polychlorinated quarterphenyl (PCQ) level was added on June 16, 1981. The study group of Yusho started to measure blood levels of dioxins in annual medical check-ups from 2001. It was considered appropriate to add an item corresponding to the blood 2,3,4,7,8-penta-chlorodibenzofurans (PeCDF) level; therefore the criteria were supplemented and further revised on September 29, 2004.** |
| --- |
|  |
| **Conditions of onset** |
| **1) Ingestion of Kanemi rice bran oil contaminated with PCBs** |
| **2) Vertical PCB transmission from mothers with Yusho to infants in some cases** |
| **3) Familial occurrence seen in many cases** |
|  |
| **Important manifestations** |
| **1. Acneform eruptions** |
| **Black comedones seen on the face, buttocks and other intertriginous sites; comedones with inflammatory manifestations; and subcutaneous cysts with atheroma-like contents that tended to suppurate.** |
| **2. Pigmentation** |
| **Pigmentation of the face, palpebral conjunctivae, gingivae, and nails etc. (including so-called ‘black babies’)** |
| **3. Hypersecretion of the meibomian glands** |
| **4. Unusual composition and concentration of PCBs in the blood** |
| **5. Abnormal level of blood PCQ** |
| **1) ≥0.1 ppb: an abnormally high concentration** |
| **2) 0.03 to 0.09 ppb: the boundary between high and normal concentrations** |
| **3) ≤0.02 ppb (detection limit): normal concentration** |
| **6. Abnormal level of blood PeCDF** |
| **1) ≥50 pg/g lipids: an abnormally high concentration** |
| **2) 30 to 50 pg/g lipids: a relatively high concentration** |
| **3) <30 pg/g lipids: normal concentration** |
|  |
| **Standard symptoms and findings** |
| **1. Subjective symptoms** |
| **1) General fatigue** |
| **2) Headaches, dull headaches** |
| **3) Paresthesia of the extremities (abnormal sensation)** |
| **4) Increased eye discharge** |
| **5) Cough and sputum** |
| **6) Constant abdominal pain** |
| **7) Altered menstruation** |
| **2. Objective findings** |
| **1) Manifestation of bronchitis** |
| **2) Deformation of nails** |
| **3) Bursitis** |
| **4) Increased neutral fat in the serum** |
| **5) Increased serum γ-glutamyl transpeptidase (γ-GTP)** |
| **6) Decrease in serum bilirubin** |
| **7) Small-for-date baby** |
| **8) Growth retardation and dental abnormality (retarded eruption of permanent teeth)** |
